# Promiscuity progression of bioactive compounds over time

**DOI:** 10.12688/f1000research.6473.1

**Published:** 2015-05-13

**Authors:** Ye Hu, Swarit Jasial, Jürgen Bajorath

**Affiliations:** 1 Department of Life Science Informatics, B-IT, LIMES Program Unit Chemical Biology and Medicinal Chemistry, Rheinische Friedrich-Wilhelms-Universität, Dahlmannstr. 2, Bonn, D-53113, Germany

**Keywords:** Polypharmacology, compound promiscuity, pharmaceutical targets, publicly available activity data, data growth, data confidence levels, promiscuity progression

## Abstract

In the context of polypharmacology, compound promiscuity is rationalized as the ability of small molecules to specifically interact with multiple targets. To study promiscuity progression of bioactive compounds in detail, nearly 1 million compounds and more than 5.2 million activity records were analyzed. Compound sets were assembled by applying different data confidence criteria and selecting compounds with activity histories over many years. On the basis of release dates, compounds and activity records were organized on a time course, which ultimately enabled monitoring data growth and promiscuity progression over nearly 40 years, beginning in 1976. Surprisingly low degrees of promiscuity were consistently detected for all compound sets and there were only small increases in promiscuity over time. In fact, most compounds had a constant degree of promiscuity, including compounds with an activity history of 10 or 20 years. Moreover, during periods of massive data growth, beginning in 2007, promiscuity degrees also remained constant or displayed only minor increases, depending on the activity data confidence levels. Considering high-confidence data, bioactive compounds currently interact with 1.5 targets on average, regardless of their origins, and display essentially constant degrees of promiscuity over time. Taken together, our findings provide expectation values for promiscuity progression and magnitudes among bioactive compounds as activity data further grow.

## Introduction

Polypharmacology is an emerging theme in pharmaceutical research and refers to the property of many bioactive compounds or drugs to act on multiple physiological targets, modulate different signaling pathways, and elicit multi-target-dependent pharmacological effects ^1– 3^. The molecular basis of polypharmacology is provided by compound promiscuity, which is defined as the ability of small molecules to specifically interact with multiple targets ^4, 5^. It should be emphasized that this form of “specificity pattern promiscuity” is distinct from non-specific interactions or assay artifacts ^6– 8^. In light of the latter problems, it is important to identify compound classes that are frequently responsible for artificial activity readouts ^7, 8^, e.g. through reactivity under assay conditions. Even in the absence of interaction artifacts, the experimental assessment of promiscuity, e.g. by systematic compound profiling on target sets or families, might be affected by assay confidence limits or detection techniques ^9^, as is the case with any screening experiment. Hence, it might sometimes be difficult to clearly distinguish between “assay promiscuity” and true target promiscuity.

In addition to experimental studies, promiscuity can also be assessed computationally by mining the rapidly increasing amounts of compound activity data that become available and systematically collecting target annotations for compounds ^3– 5^. For computational analysis, it is also of critical importance to carefully consider activity data integrity and confidence levels to arrive at reliable promiscuity estimates ^5^. For compound data mining, public repositories are essential including ChEMBL ^10^, the major public source of data from medicinal chemistry, PubChem’s BioAssay database ^11^, the major source of screening data, and DrugBank ^12^, which collects target annotations for drug candidates and drugs. Systematic computational analysis of promiscuity has been largely dependent on these resources (although proprietary pharmaceutical data have also been used).

In recent years, computational investigations have provided different promiscuity estimates, depending on the specific aims, study design, and data selection criteria that were applied. Drugs have been the major focal point of these studies. Early estimates on the basis of drug-target networks have suggested that a drug interacts with two targets on average ^13^. Recently, it has been proposed that drugs directed against different target families bind to an average of two to seven targets, depending on their primary target families, and that more than 50% of current drugs bind to more than five targets ^3^. For bioactive compounds, analysis of high-confidence activity data indicated that they interact with an average of one to two targets, with most promiscuous compounds being annotated with two to five targets from the same target family ^5, 14^. Moreover, the analysis of high-confidence activity data from 1085 PubChem confirmatory bioassays for 439 targets revealed that a confirmed hit interacted with only two targets on average, although nearly 80% of these active PubChem compounds were tested in more than 50 different assays ^15^. Taken together, computational analyses of bioactive compounds from medicinal chemistry and screening sources indicated the presence of lower degrees of promiscuity overall than was detected for drugs.

These findings could be rationalized based on the assumption that drugs might often be more extensively tested against different targets than average bioactive compounds. However, this would not explain the relatively low degree of promiscuity observed for active compounds from screening libraries, many of which are extensively tested. Furthermore, promiscuity estimates from computational analysis are occasionally questioned in light of data sparseness ^16^, referring to the fact that available active compounds have not been tested against all targets, which represents the vision and ultimate goal of chemogenomics ^17^. Data incompleteness might principally lead to an underestimation of the degree of promiscuity. However, it remains unclear how significant such deviations might be. In fact, if one considers that millions of activity annotations are already available at present, it should be possible to deduce statistically meaningful trends from such large data samples. Such promiscuity trends might be detected by monitoring promiscuity over time as activity data grow. In a recent study, this type of analysis has been carried out for approved drugs ^18^. For a set of 518 drugs, promiscuity was quantified over different time intervals considering activity data at different confidence levels. When only high-confidence activity records were considered, an increase in the average degree of promiscuity from 1.5 to 3.2 targets per drug was detected over a period of 14 years (from 2000 and 2014). By contrast, when all available activity data were considered, regardless of confidence levels, partially unrealistic increases in promiscuity were observed, ranging from six targets per drug on average in 2000 to more than 28 targets in 2014 ^18^. For individual high-profile drugs, literally hundreds of target annotations were detected when no confidence criteria were applied. This study showed how dramatic the influence of data confidence levels on promiscuity assessment could be. Furthermore, when considering the results obtained on the basis of high-confidence activity data, the findings also corroborated conclusions drawn from earlier studies discussed above, which indicated that detectable promiscuity of active compounds and drugs might be lower overall than often assumed (and that these observations might not be largely determined by data incompleteness).

To further refine current promiscuity estimates, we report herein a detailed analysis of the degree of promiscuity of current bioactive compounds monitored over time, spanning a period of 39 years. Special attention was paid to compounds that were first recorded many years ago and are still available. Promiscuity was viewed in light of data growth and monitored using high- and low-confidence activity data. A large number of compounds qualified for this analysis and clear trends were detected. The results of our analysis are presented in the following.

## Materials and methods

### Growth of compound activity data

The ChEMBL database ^10^ that was analyzed collects large numbers of compounds and activity data, mainly from the medicinal chemistry literature and the PubChem BioAssay database ^11^. The current ChEMBL version (v.20) contains 1,463,270 structurally distinct compounds with activity against 10,774 targets. From 1,148,942 assays, a total of 13,520,737 activity records originated, as reported in Table 1. To systematically explore data growth over time, our analysis focused on data for which release dates were available, which included 913,972 compounds, 10,142 targets, 872,577 assays, and 5,258,052 activity records ( Table 1). The growth of these data was monitored on an annual basis. For each year, the number of new entries that became available and the total (cumulative) number of entries was recorded.

**Table 1.** ChEMBL v.20 statistics. For ChEMBL v.20 and subsets for which specific release dates were available, the total number of compounds, targets, assays, and activity records (activities) is shown.

Number of	Total	With release dates
**Compounds**	1,463,270	913,972
**Targets**	10,774	10,142
**Assays**	1,148,942	872,577
**Activities**	13,520,737	5,258,052

### Data sets of varying confidence levels

In order to investigate compound promiscuity over time as well as the effect of data confidence levels on promiscuity degrees, two data sets with different confidence were assembled from ChEMBL v.20. For the high-confidence data set, a series of selection criteria was applied. Compounds with direct interactions (i.e. assay relationship type “D”) with human single-protein targets at the highest confidence level (i.e. assay confidence score 9) were collected. The two ChEMBL parameters ‘assay relationship type’ and ‘assay confidence score’ qualitatively and quantitatively describe, respectively, the level of confidence that the activity against a given target is evaluated in a relevant assay system. Accordingly, type “D” and score 9 represent the highest level of confidence for activity data. In addition, two types of activity measurements were considered; assay-independent equilibrium constants (K _i_ values) and assay-dependent IC _50_ values. To ensure a high level of data integrity, only compounds with explicitly defined K _i_ and/or IC _50_ values were selected. Hence, approximate measurements such as “>”, “<”, and “~” were disregarded. Furthermore, activity records including the comments “inactive”, “inconclusive”, or “not active”, were discarded. Thus, this compound set exclusively contained high-confidence activity data. By contrast, the low-confidence data set comprised all compounds with reported interactions against human single-protein targets, regardless of their confidence levels and activity measurement types.

### Monitoring compound activity records over time

On the basis of the high- and low-confidence data sets, the progression of compound promiscuity was quantified. Activity records with release dates were assigned to individual compounds. For each year, activity records were assembled. For instance, if a compound was reported to be active against target A in 1990, targets B and C in 2000, and target D in 2005, the cumulative activity records for this compound consisted of target A in 1990, targets A, B and C in 2000, and targets A, B, C, and D in 2005. Thus, the degree of promiscuity of this compound increased from 1 over 3 to 4. For a given year, the average degree of promiscuity was calculated over all qualifying compounds. In addition, subsets of compounds for which activity data first became available in 1994 (20 year activity history) or 2004 (10 year history) were separately monitored.

## Results and discussion

### Growth of compounds, targets, assays, and activity records

**Figure 1.**
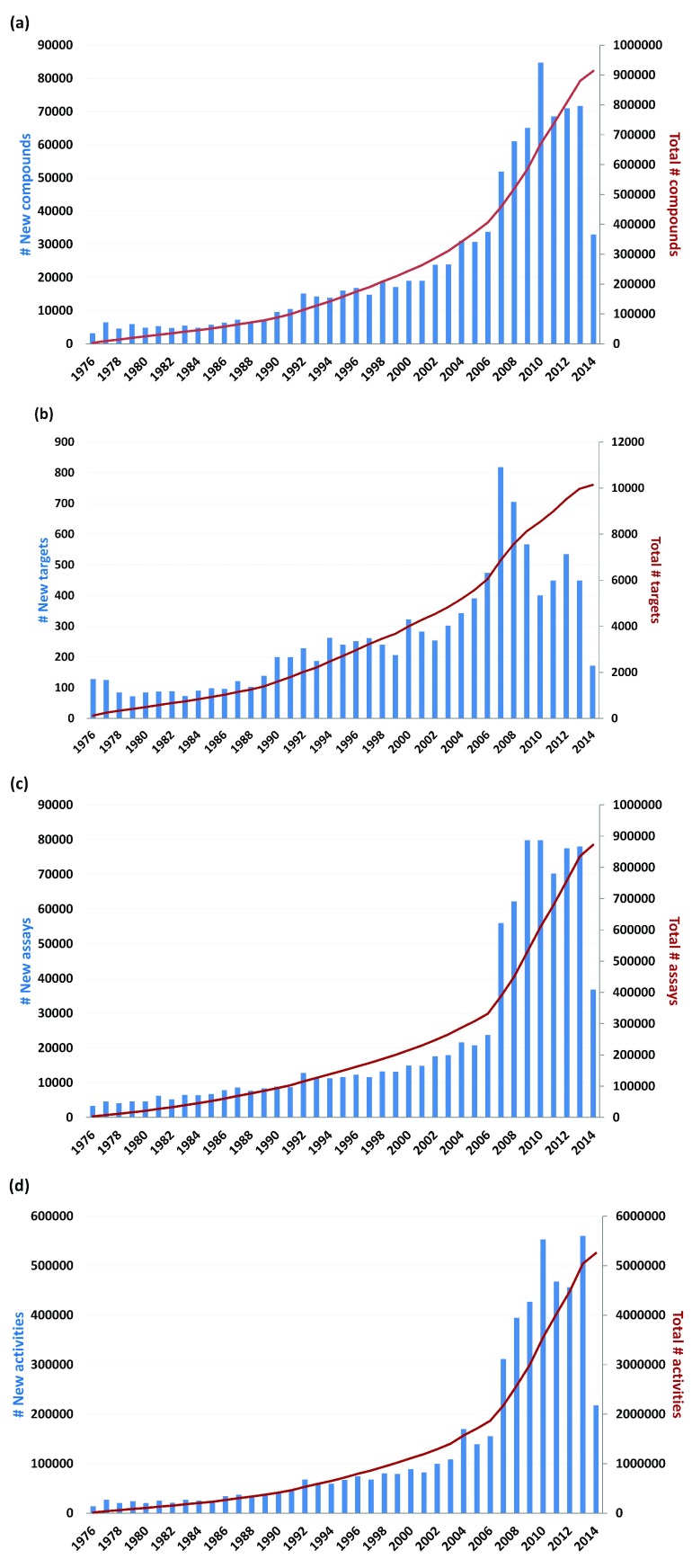
Growth of compounds, targets, assays, and activity records. The growth of compounds ( **a**), targets ( **b**), assays ( **c**), and activity records ( **d**) is reported. In ( **a**), the number of new compounds becoming available each year is provided using blue bars (scale on the left vertical axis) and the cumulative number of compounds is given as a red line (scale on the right). Corresponding representations are used in ( **b**)–( **d**).

In ChEMBL v.20, release dates were reported for 913,972 compounds, 10,142 targets, 872,577 assays, and 5,258,052 activity records ( Table 1). Initially, the growth of these source data was analyzed over time. Figure 1 reports the number of new entries that became available each year since 1976 and the total (cumulative) number of entries for each year. As shown in Figure 1a, only 3188 compounds were reported in 1976. In 1977, 6496 compounds were released, yielding a total of 9684 compounds. Since then steady growth in compound numbers was observed until 2006 when the growth rate became nearly exponential, with ~50,000–80,000 compounds becoming available in 2007 and subsequent years. The number of compounds released in 2014 was much lower, probably due to the likely situation that not all new compounds and activity data published in 2014 would have been deposited in the database by the end of the year. Similar growth trends were observed for targets ( Figure 1b), assays ( Figure 1c) and activity records ( Figure 1d).

In Table 2, the numbers of compounds, targets, assays, and activity records available in 1976 and 2014 are compared. Within this 39-year period, available activity records increased most significantly from 13,999 to 5,258,052 (by a factor of ~376). For compounds and assays, growth factors were comparable (~287 and ~261, respectively). The number of targets increased by a factor of ~79.

**Table 2.** Data growth. The numbers of compounds, targets, assays, and activity records available in 1976 and 2014 are compared.

Number of	1976	2014	Increase (fold)
**Compounds**	3188	913,972	286.7
**Targets**	128	10,142	79.2
**Assays**	3347	872,577	260.7
**Activities**	13,999	5,258,052	375.6

Overall, significant increases in the number of compounds, targets, assays, and activity records were observed, especially from 2007 on, thus providing a sound basis for the analysis of compound promiscuity progression over time.

### High- and low-confidence data sets

Based on the selection criteria detailed above, two sets of compounds with high- and low-confidence activity data were assembled. In the low-confidence set, compounds with any reported activities against human single-protein targets were included, without applying additional data confidence criteria. By contrast, for the high-confidence set, additional criteria were applied including assay confidence levels as well as the type and integrity of potency measurements. As reported in Table 3, the high-confidence set contained 154,062 compounds active against 1449 targets, yielding a total of nearly 234,000 activity records with release dates. In the low-confidence set, 361,159 compounds active against 2552 targets were available, yielding a total of nearly 782,000 activity records. Data sets of this magnitude were expected to reveal statistically relevant trends in promiscuity progression.

**Table 3.** Data with different confidence levels. The numbers of compounds, targets, assays, and activity records with available release dates are reported for the high- and low-confidence data sets, respectively.

Number of	High-confidence set	Low-confidence set
**Compounds**	154,062	361,159
**Targets**	1449	2552
**Assays**	27,876	141,319
**Activities**	233,971	781,707

### Compound promiscuity over time

**Figure 2.**
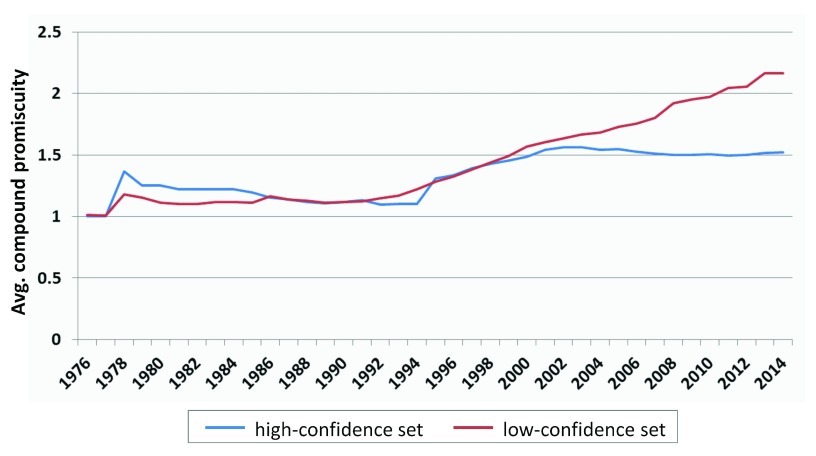
Compound promiscuity over time. For compounds in the high- and low-confidence data sets, the average degree of compound promiscuity is reported over different years.

***Global estimate.*** For compounds in the high- and low-confidence data sets, the average degree of compound promiscuity was determined over the years, as reported in Figure 2. Early on, compounds from both data sets were mostly associated with single-target activities (corresponding to a promiscuity degree of 1). Beginning in 2004, a difference in promiscuity between the high- and low-confidence sets became apparent. However, only a limited increase in promiscuity was observed for compounds from both data sets. From 1976 to 2014, the average degree of promiscuity increased from 1 to 1.5 for the high- and from 1 to 2.2 for the low-confidence data set, thus indicating an overall low degree of promiscuity among bioactive compounds. More interestingly, the average degree of promiscuity for compounds in the high-confidence set only increased by 0.4 (i.e. by less than one target) after 1994 and essentially remained constant between 2004 and 2014, although the amount of available compounds and activity data dramatically increased after 2006 ( Figure 1).

**Figure 3.**
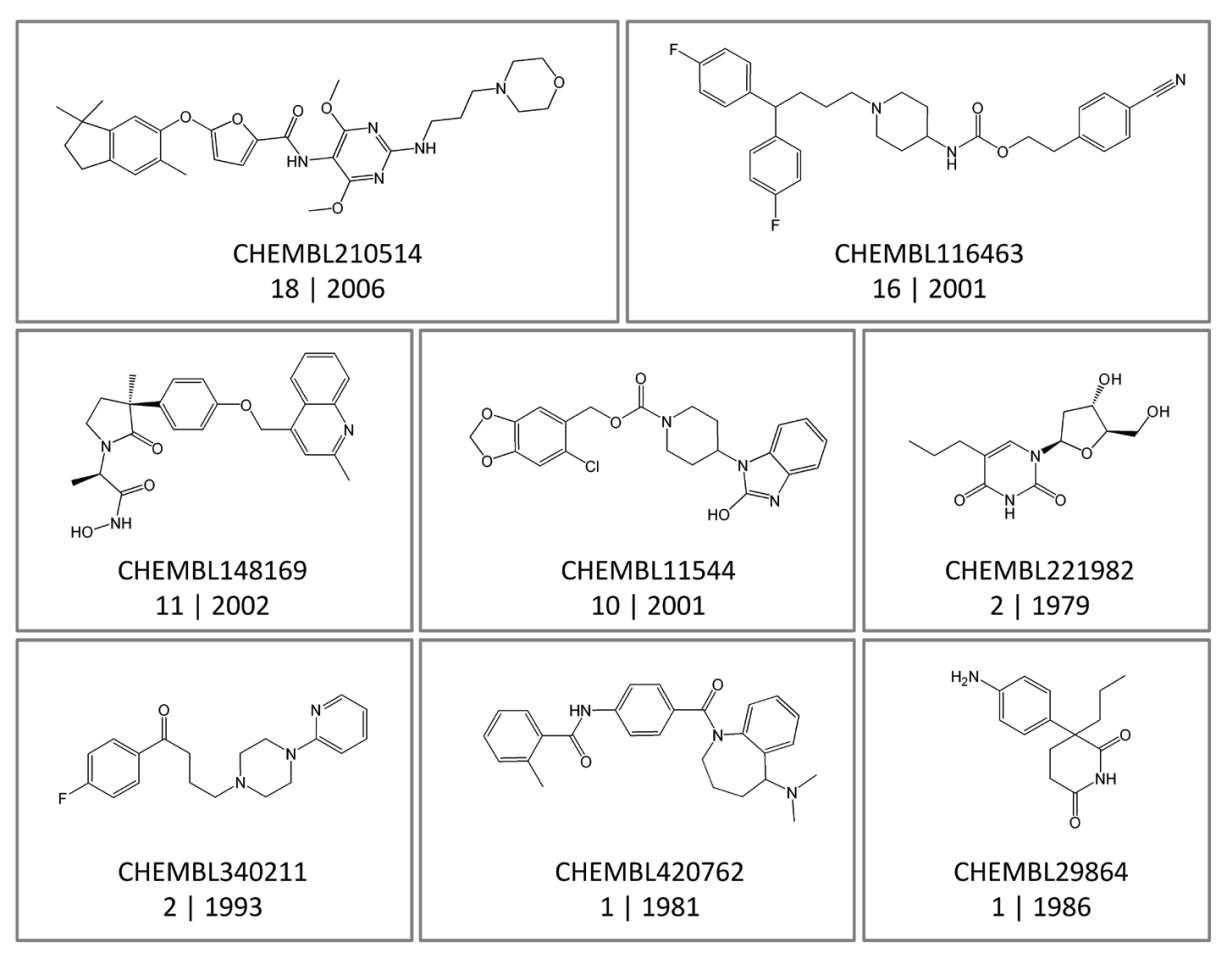
Compounds with constant promiscuity. Shown are eight exemplary compounds from the high-confidence data set that displayed a constant degree of promiscuity over different time periods. For each compound, its ChEMBL ID, the degree of promiscuity, and the first year in which target-specific activities were reported are given. For example, “2 | 1993” (lower left) indicates that this compound was first reported in 1993 to be active against two targets and that this degree of promiscuity (i.e., 2) has remained constant until 2014.

***Promiscuity on a per-compound basis.*** In addition to the global assessment of compound promiscuity, progression of promiscuity was also monitored for individual compounds. Table 4 reports the number of compounds with increasing degrees of promiscuity over time. Strikingly, a total of 151,786 (i.e. 98.5%; high-confidence set) and 352,466 (97.6%; low-confidence set) compounds displayed constant degrees of promiscuity over time. Exemplary compounds are shown in Figure 3. These compounds were active against varying numbers of targets. Yet their degrees of promiscuity remained constant until 2014. It is unlikely that subsets of large numbers of compounds with a constant degree of promiscuity over many years have not been tested in various assays. For example, the compound shown at the bottom left in Figure 3 ( CHEMBL340211) was reported to be active against two targets in 1993. However, no additional high-confidence activity data became available for this compound during the following 21 years. An abundance of such examples exists for compounds active across current targets.

**Table 4.** Increasing promiscuity. The number of compounds with increasing degrees of promiscuity (∆Promiscuity) is reported for the high- and low-confidence data sets. For example, “0” indicates that the degree of promiscuity remained constant over time and “5” that the degree of promiscuity increased by five target annotations.

∆Promiscuity	#Compounds	
	High-confidence set	Low-confidence set
**0**	151,786	352,466
**1**	1239	4099
**2**	469	1721
**3**	220	816
**4**	102	398
**5**	65	305
**6–10**	130	698
**11–20**	40	283
**21–50**	9	137
**> 50**	2	236
**Total**	154,062	361,159

Increases in promiscuity were only observed for 2276 and 8693 compounds in the high- and low-confidence sets, respectively ( Table 4). Moreover, only 181 (high-confidence set) and 1354 (low-confidence set) compounds - a minute fraction of all monitored compounds - gained more than five target annotations over the years.

**Figure 4.**
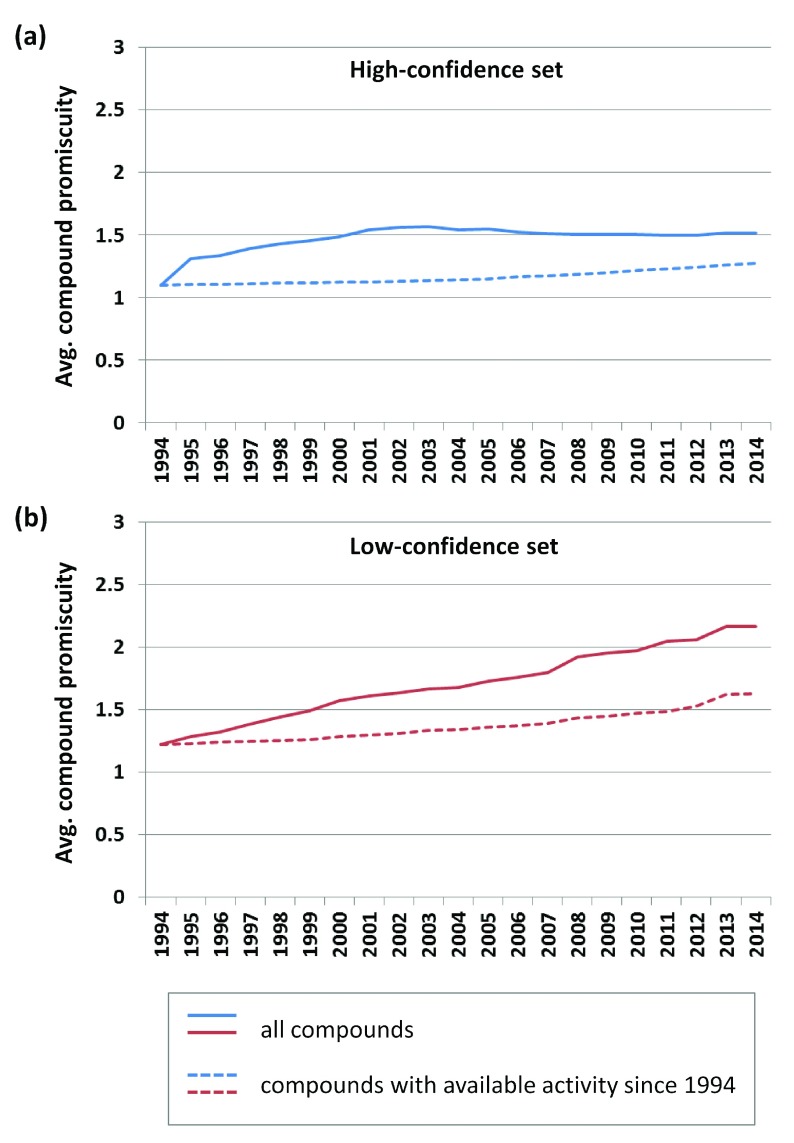
Promiscuity of compounds available since 1994. The average degree of promiscuity was compared for all high- ( **a**) and low-confidence ( **b**) set compounds (solid lines) and subsets of compounds reported to be active beginning in 1994 (dashed lines).

***Compounds with 20 year activity history.*** Subsets of compounds reported to be active since 1994 were assembled. From the high- and low-confidence sets, 1040 and 19,351 qualifying compounds were obtained, respectively. Promiscuity progression over the subsequent 20 years was separately analyzed for these compound subsets. Figure 4a shows that the degree of promiscuity of the 1040 compounds from the high-confidence data set essentially remained constant, with an increase from 1.1 (1994) to only 1.2 (2014), hence representing lower promiscuity than the global degree of promiscuity determined for the high-confidence set. For the 19,351 compounds from the low-confidence set, the degree of promiscuity only increased from 1.3 to 1.6, which was also lower than the global degree of promiscuity for this set ( Figure 4b). Hence, on the basis of activity data monitored over the course of 20 years, compound promiscuity only slightly increased and promiscuity rates were lower than might have been anticipated, although large amounts of activity data became available over time.

### Current promiscuity levels for bioactive compounds

Up-to-date promiscuity levels were determined for all qualifying compounds, the subsets of compounds for which activity data first became available in 1994 (20 year activity history), and compound subsets for which activity data first became available in 2004 (10 year history). The results are reported in Table 5. The degree of promiscuity was consistently low in all cases and differences in promiscuity were only marginal. For the high-confidence set, the average degree of promiscuity ranged from 1.3 (20 year activity history) over 1.5 (all compounds) to 1.7 (10 year activity history). For the low-confidence set, it ranged from 1.6 (20 year history) over 2.0 (10 year history) to 2.2 (all compounds). Thus, bioactive compounds generally displayed only a low degree of promiscuity, regardless of the data set from which they originated.

**Table 5.** Current promiscuity rates. For the high- and low-confidence data sets, the current average degree of promiscuity is reported for all compounds and compound subsets with activity records available since 1994 and 2004, respectively.

		Avg. promiscuity rate
**High-confidence** **set**	All 154,062 compounds	1.5
	1040 compounds with activity available since **1994**	1.3
	9979 compounds with activity available since **2004**	1.7
**Low-confidence** **set**	All 361,159 compounds	2.2
	19,351 compounds with activity available since **1994**	1.6
	101,370 compounds with activity available since **2004**	2.0

## Conclusions

Currently available activity data provide an unprecedented source of information for the analysis of bioactive compounds. To assess the promiscuity of bioactive compounds in detail, available activity data have been assigned on the basis of release dates to individual years, thus enabling the study of data growth and compound promiscuity on a time scale and in context. Monitoring compound promiscuity over time was expected to reveal sound trends concerning promiscuity progression and evolving magnitudes. Furthermore, to take data confidence explicitly into account, high- and low-confidence compound data sets were separately generated and analyzed. Data growth and promiscuity progression were ultimately monitored over nearly 40 years (beginning in 1976), both at a global level, as well as focusing on individual compounds or compound subsets of compounds (from the high- and low-confidence sets) with a 20 year or 10 year activity history. The analysis provided a perhaps unexpectedly clear picture and revealed generally low degrees of promiscuity for bioactive compounds, regardless of their activities and origins. Moreover, only minor increases in promiscuity over time were detected for compounds from all sets and subsets, although activity data dramatically increased since 2007. For the high-confidence set, the average degree of promiscuity only increased from 1 to 1.5 over time. Furthermore, even for the low-confidence set, an increase in the degree of promiscuity to only 2.2 was detected. Interestingly, in both cases, promiscuity was constant over time for most compounds. Moreover, for the high-confidence set, the degree of promiscuity essentially remained constant between 2004 and 2014, despite massive data growth. Given the extensive time course followed, the large data volumes accumulated, and the consistent trends detected, these findings could hardly be solely attributed to data incompleteness (although conclusions drawn from data mining might well be affected by data integrity and/or sparseness issues). In our systematic analysis, bioactive compounds were found to display only low degrees of promiscuity, with a surprisingly small influence of data confidence levels, and very limited promiscuity progression over time. The observed trends are anticipated to remain stable as compounds and activity data continue to grow at high rates and provide reference points for future studies of compound and drug promiscuity as the molecular basis of polypharmacology.

## Data availability

The data selection criteria specified in the Materials and methods section make it possible to reproduce all data sets from ChEMBL v.20, including release dates. The resulting data set statistics are provided in the first part of the Results and discussion section.
