# Supplementary material for: Promiscuity progression of bioactive compounds over time
Source: F1000Res. 2015 May 13;4(Chem Inf Sci):118. [Version 1] doi: 10.12688/f1000research.6473.1 (PMC4448747; doi:10.12688/f1000research.6473.1)
Supplement: Peer review history

# Peer review: Referee response for version 1

**DOI:** 10.5256/f1000research.6945.r8802

**Published:** 2015-05-29

**Authors:** John Lowe

**Affiliations:** 1 JL3Pharma LLC, Stonington, CT, USA

The authors investigate the potential growth of off-target activity over time as new assays become available.  They control for multiple potential confounds, and possibly the most important is data quality enabling confidence in the results.  They note that prior data indicated screening compounds typically bind to at least two targets, while drugs may bind up to seven, but this result might be skewed by insufficient data quality.  Their study reported here uses very large datasets and controls for the growth of compound number as well as new data over time.  They also deliberately analyze high- and low-quality (or confidence) data separately.  This careful analysis gives them a much clearer picture of the changes in compound promiscuity over time, and reveals a low level of off-target activity and only a slight increase with time.  Despite new assays becoming available at an increasing rate, compound promiscuity has not increased significantly, a result that will surprise many readers, but which the authors have documented admirably.  I highly recommend this manuscript for indexing.

I have read this submission. I believe that I have an appropriate level of expertise to confirm that it is of an acceptable scientific standard.

# Peer review: Referee response for version 1

**DOI:** 10.5256/f1000research.6945.r8663

**Published:** 2015-05-26

**Authors:** Christopher Southan

**Affiliations:** 1 IUPHAR/BPS Guide to PHARMACOLOGY, Center for Integrative Physiology, University of Edinburgh, Edinburgh, UK

The analysis presented in this paper is of considerable interest and should be indexed, However, I think there are many confounding factors within the ChEMBL data that the authors have not addressed sufficiently.  I will pick up some of these below.

1. Polypharmacology usually implies the affects mediated via the multiple targets are therapeutically “positive”.  Is this the authors' implication also?  Otherwise the term implicitly extends to toxicity and side effects.
2. Figure 1 should include the distribution that underlies the other three, namely papers per-year.
3. While the databases used were different, a published tracking of compound output from papers showed much less increase over 20 years than in figure 1 (PMID:24204758) although the target growth pattern was similar.  Have the authors checked that ChEMBL did not pick up new journal coverage from 2008 that would spike the increases?
4. I would like more detail on how the filtration methodology in the paper is used to extract and score (a flow chart would help).  Let me pose a hypothetical case of two compounds. The first ranks target A at an IC50 of 20nM and target B at 30 nM. The second compound is 1nM and 500 nM for the same two targets.  Do the two cases get the same promiscuity score?  (It would be confounding if they did.)
5. What happens when compound-target-assay values are identical for different publication years (not uncommon in ChEMBL) - Do you score only the first year ?
6. I’m confused by use of  “release date” (as for a database)  surely “publication date”  is meant? 
7. For fig.3  I suggest  the dominant  explanation for apparently constant promiscuity is simply “publish-and-forget” (i.e. researchers typically do not re-test compounds published by others).  As we know re-testing leading to the publication of new results (promiscuous or not) will be largely dependent as to whether structures become reference compounds, are advanced into development, or become drugs. So could the “papers-per-compound” relationship be plotted to provide insight into this?
8. There are other confounding trends that could be tested for, for example targets-per-paper (i.e. < cross-screening over the years might correlate with apparent promiscuity <) and orthologue vs paralogoue cross screening (i.e. if the average human:rodent ratio changes over time for the low confidence set).
9. Why not select kinase inhibitors as a control subset? We would expect these to exhibit highest promiscuity and they would thus be an important methodological cross-check. 
10. In terms of other obvious hypothesis checks why not split by LogP (as might increase promiscuity) and Mw (as might decrease it) ?
11. While appreciating the academic imperative I do wish this team could have merged some of their previous papers that appear to address essentially the same theme.  For example, comparing drug (ref.18) vs non-drug promiscuity in the same standardised study is better (easier to review even :) than splitting the result sets.

I have read this submission. I believe that I have an appropriate level of expertise to confirm that it is of an acceptable scientific standard, however I have significant reservations, as outlined above.

# Peer review: Referee response for version 1

**DOI:** 10.5256/f1000research.6945.r8662

**Published:** 2015-05-14

**Authors:** Georgia B. McGaughey

**Affiliations:** 1 Vertex Pharmaceuticals, Boston, MA, USA

I believe this paper should be indexed as I have not seen such a methodical and quantified examination of promiscuity before.  The article is well written and easy to read.  Figures are compelling.

Although there is compelling data included suggesting that over time, promiscuity generally doesn’t increase for a given compound, I'm a bit skeptical on concluding that promiscuity may not have markedly increased over the past few decades based on merely ChEMBL. I, however, recognize that those in academia (or in a biotech company where large receptor screening may not be part of the business model) may not have access to an orthogonal data set.  Frankly, other than ChEMBL, I’m not sure where else one would go to look for off target data.  There are purchasable databases (e.g. Integrity).  However, more public data is relatively sparse.  Even a (young) small-ish biotechnology company would not have enough data to utilize.

One topic that has been raised in the literature the past few years is the concept of phenotypic versus target based drug discovery approaches to developing new medicines.  I would have liked to see some differentiation between promiscuity of target based versus phenotypic based projects.  Is that something the reviewers can go back to and annotate their data set? 

Additionally, discussion around the differences between promiscuity and polypharmacology should be elaborated upon.  I realize this is raised in the "introduction", but I would have liked to see more attention paid to this topic.  

Finally, will the data sets be publicly available with annotations?

I have read this submission. I believe that I have an appropriate level of expertise to confirm that it is of an acceptable scientific standard.
